# Maßnahmen gegen Einsamkeit – Beispiele aus einer international vergleichenden Perspektive

**DOI:** 10.1007/s00103-024-03945-y

**Published:** 2024-08-29

**Authors:** Claus Wendt

**Affiliations:** Lehrstuhl für Soziologie der Gesundheit und des Gesundheitssystems, Universität Siegen, Adolf-Reichwein-Str. 2, 57068 Siegen, Deutschland

**Keywords:** Internationaler Vergleich, Einsamkeit, Gesundheit, Nachbarschaft, Wohlfahrtsstaat, International comparison, Loneliness, Health, Neighborhood, Welfare state

## Abstract

Das Ausmaß von Einsamkeit unterscheidet sich im internationalen Vergleich. Vor allem in den entwickelten Wohlfahrtsstaaten Nordeuropas sind die Einsamkeitswerte in der Bevölkerung vergleichsweise gering. Der Anstieg an Einsamkeit in vielen Ländern zeigt allerdings, dass bestehende Konzepte für einen wirksamen Schutz vor Einsamkeit nicht ausreichen. Auch jüngere Menschen müssen gestärkt werden, damit sie sich bei Ausgrenzungserfahrungen und Einsamkeit nicht zurückziehen. Hierfür werden qualifizierte Fachkräfte in Kindergärten, Schulen, Vereinen und weiteren Freizeiteinrichtungen benötigt, die Kindern und Jugendlichen helfen, soziale Kontakte aufzubauen. Bei den Strategien gegen Einsamkeit haben sich Freundschafts- und Nachbarschaftsmodelle als erfolgreich erwiesen. Eine höhere Identifikation mit der Nachbarschaft und ein Gefühl der Sicherheit tragen zum Aufbau sozialer Netzwerke bei und reduzieren die Einsamkeit. Für ältere Menschen ist es wichtig, dass sie in ihrem gewohnten sozialen Umfeld wohnen bleiben und ihre sozialen Kontakte aufrechterhalten können. Hierfür sind erreichbare öffentliche Plätze mit Pflegeangeboten und Unterstützungsleistungen zu verbinden, die auf die Bedürfnisse und Wünsche älterer Menschen zugeschnitten sind.

## Abstract (en)

The extent of loneliness differs internationally. Especially in the developed welfare states of Northern Europe, loneliness levels in the population are comparatively low. However, the increase in loneliness in many countries shows that existing concepts are not sufficient to provide effective protection against loneliness. Younger people also need to be strengthened so that they do not withdraw when they experience social exclusion and loneliness. This requires qualified specialists in kindergartens, schools, sports clubs, and other leisure facilities who help children and young people to establish social contacts. Friendship and neighborhood models have proven to be successful when it comes to strategies to combat loneliness. Greater identification with the neighborhood and a feeling of security help build social networks and reduce loneliness. For older people it is important to be able to stay in their usual social environment and maintain their social contacts. To this end, accessible public spaces must be combined with home care and support services that are tailored to the needs and wishes of older people.

## Einleitung

Mit Einsamkeit sind individuelle und gesellschaftliche Risiken wie Krankheit, eine sinkende Solidarität, ein sinkendes Vertrauen in Mitmenschen und in politische und gesellschaftliche Institutionen sowie eine Schwächung der Innovationskraft auf gesellschaftlicher, politischer und wirtschaftlicher Ebene verbunden [1–5]. Da Menschen unter Einsamkeit leiden, wird eine Reaktion auf das gesellschaftliche Problem der Einsamkeit als wichtig angesehen. In diesem Beitrag werden Konzepte gegen Einsamkeit aus einer international vergleichenden Perspektive vorgestellt und analysiert [1–3]. In international vergleichenden Studien wird Einsamkeit häufig als eine wahrgenommene Diskrepanz zwischen den gewünschten und tatsächlichen sozialen Beziehungen definiert [3, 6]. Dieser Definition wird in diesem Beitrag gefolgt.

Gesellschaftliche Gruppen leiden unterschiedlich stark unter Einsamkeit. Bei sozial Schwachen und Alleinerziehenden ist die Einsamkeit überdurchschnittlich hoch [4]. Im Alter steigt häufig die Einsamkeit und ist dann für einen erheblichen Teil der jeweiligen Altersgruppen eine hohe Belastung [7]. Die politischen Reaktionen auf die Gefahr der Einsamkeit hängen auch mit 2 jüngeren Erfahrungen zusammen. Die Covid-19-Krise hat in vielen Ländern gezeigt, dass politische Maßnahmen zur Eindämmung der Pandemie gravierende Folgewirkungen hatten, zu denen auch ein Anstieg der Einsamkeit zählt. Außerdem leiden auch junge Menschen häufiger unter Einsamkeit [1, 8, 9], deren Entwicklungschancen dadurch beeinträchtigt werden können.

Im Folgenden werden zunächst ausgewählte Unterschiede der Einsamkeit im internationalen Vergleich dargestellt. Dabei zeigt sich, dass Länder, in denen soziale Probleme mithilfe solidarisch finanzierter Sicherungssysteme reduziert werden, geringere Einsamkeitswerte aufweisen. Diese und weitere institutionelle Regelungen werden in diesem Beitrag als indirekte Maßnahmen gegen Einsamkeit bezeichnet, da sie zum Schutz gegen andere soziale Risiken und nicht explizit zur Bekämpfung von Einsamkeit aufgebaut wurden. Indirekte Maßnahmen gegen Einsamkeit werden im dritten Abschnitt diskutiert. Anschließend erfolgt ein Überblick über Beispiele für direkte Maßnahmen, die eingeführt wurden, um das Problem der Einsamkeit zu bekämpfen. Einige Konzepte gegen Einsamkeit wurden wissenschaftlich evaluiert, die ebenfalls dargestellt und in der abschließenden Diskussion eingeordnet werden.

## Einsamkeit im internationalen Vergleich

Es zeigt sich wiederholt, dass in Europa vor allem nordeuropäische Länder vergleichsweise niedrige Einsamkeitswerte aufweisen [4, 7]. In internationalen Erhebungen, die das Ausmaß der Einsamkeit erfassen, wird i. d. R. die Frage gestellt, wie häufig sich die Befragten in der vergangenen Woche einsam gefühlt haben (mit den Antwortoptionen „nie oder fast nie“, „manchmal“, „meistens“, „immer oder fast immer“; [4]). Die im Folgenden aufgeführten Länderunterschiede wurden in Studien mit neueren Daten weitgehend bestätigt [10]. In Dänemark, Norwegen und in den Niederlanden liegen die Werte in der mittleren Altersgruppe zwischen 2 % und 3,5 %. Deutlich höher sind die Zahlen in Südeuropa. Hier wurden niedrige Einsamkeitswerte vermutet, da die Großfamilie nach wie vor eine erhebliche Rolle spielt und der Familienzusammenhalt als hoch gilt. In Spanien und Portugal liegen die Werte im mittleren Alter zwischen 6 % und 9 %. Es liegt vor allem an der Qualität der Familienbeziehungen, ob Familienmitglieder sich einsam fühlen oder nicht. Bei schlechten Beziehungen ist auch eine eng miteinander verbundene Großfamilie kein Schutz, sondern kann im Gegenteil zu Einsamkeit beitragen [11]. Besonders hohe Einsamkeitswerte sind in osteuropäischen Ländern zu verzeichnen. In Deutschland und anderen westeuropäischen Ländern liegen die Werte zwischen diesen Extremen, während die Niederlande und die Schweiz mit den vergleichsweise niedrigen Einsamkeitswerten der führenden nordischen Länder mithalten (Tab. 1).

**Tab. 1:** Einsamkeit in unterschiedlichen Altersgruppen im internationalen Vergleich [4] (2011)

Land	Anteil der Einsamkeit in %		
	Unter 30 Jahre	30–59 Jahre	60 Jahre und älter
Ukraine	15,3	19,8	34,0
Ungarn	9,6	13,3	21,1
Rumänien	11,5	10,7	18,8
Slowakei	8,8	10,5	19,6
Portugal	6,5	9,0	14,9
Spanien	4,4	6,5	11,5
Frankreich	8,2	8,8	11,4
Österreich	9,5	6,4	10,5
Belgien	6,2	6,5	8,7
England	6,3	5,5	7,4
Deutschland	5,1	4,4	7,0
Niederlande	3,4	3,3	6,0
Norwegen	2,2	2,6	5,0
Schweiz	1,3	2,6	4,8
Dänemark	3,4	1,9	3,2

Studien zeigen, dass Einsamkeit ein Problem für unterschiedliche soziale Gruppen und für alle Altersgruppen ist. Luhmann und Hawkley [12] weisen darauf hin, dass im Lebensverlauf die Einsamkeit an 2 Zeitpunkten besonders hoch ist: in Deutschland im Alter von etwa 30 und im Alter von etwa 60 Jahren. Die Einsamkeit ist im Alter von etwa 40 und etwa 75 Jahren vergleichsweise niedrig und steigt ab dem Alter von 75 Jahren wieder an [12]. Studien mit SHARE-Daten (Survey of Health, Ageing and Retirement in Europe) bestätigen die Vorteile der nordischen Länder, der Niederlande und der Schweiz in der höheren Altersgruppe [10]. Im internationalen Vergleich zeigen sich auch bei jüngeren Menschen die niedrigsten Einsamkeitswerte in entwickelten Wohlfahrtsstaaten wie Dänemark, Norwegen oder den Niederlanden. Überdurchschnittlich hohe Werte finden sich bei dieser Altersgruppe in Großbritannien und den USA, die zu den liberalen Wohlfahrtsstaaten zählen. Besonders hohe Werte sind in osteuropäischen Ländern, wie z. B. Bulgarien, zu verzeichnen, die als rudimentäre Wohlfahrtsstaaten gelten [1, 13].

Es wurde sowohl nachgewiesen, dass Einsamkeit erhebliche negative Folgen für die Gesundheit hat, als auch, dass ein schlechter Gesundheitszustand zu einer höheren Einsamkeit führt. Ein Schutz vor Einsamkeit stärkt die Gesundheit und reduziert die Sterblichkeit [14, 15]. Zu den gesundheitlichen Folgen von Einsamkeit zählen ein hoher Blutdruck, ein geschwächtes Immunsystem, ein höheres Infektionsrisiko, Herzerkrankungen, kognitive Einschränkungen, Depressionen, Suizid, Alkoholismus und Schlafprobleme [15, 16]. Die gesundheitlichen Risiken steigen an, je häufiger Menschen einsam sind. Vor allem bei chronischer Einsamkeit erhöht sich die Sterblichkeit [17]. Die enge Verbindung zwischen Einsamkeit und sozialen und gesundheitlichen Problemen zeigt, wie wichtig es ist, umfassende politische und gesellschaftliche Maßnahmen gegen Einsamkeit zu etablieren.

## Indirekte Maßnahmen gegen Einsamkeit

Die vergleichsweise niedrigen Einsamkeitswerte in ausgebauten Wohlfahrtsstaaten verweisen auf die hohe Bedeutung von sozialpolitischen Leistungen und weiteren Maßnahmen zur Förderung von Entwicklungschancen (Bildungssystem) und des sozialen Zusammenhalts (ehrenamtliches Engagement, Vereinstätigkeiten), um Einsamkeit wirksam zu bekämpfen [18]. Kirchen und Religionsverbände bilden ebenfalls wichtige Gemeinschaften, die Einsamkeit reduzieren können [19]. Im Folgenden werden ausgewählte Leistungen dargestellt, die einen sozialen Schutz bieten und Menschen in eine Gesellschaft integrieren und dadurch vor Einsamkeit schützen können. Auch wenn finanzielle Transferleistungen bei Armut und im Alter ebenfalls eine hohe Bedeutung haben, ist der Fokus auf soziale Dienstleistungen ausgerichtet.

### Kindergärten.

Die Einsamkeit hat unter den jüngeren Mitgliedern der Gesellschaft zugenommen. Kontaktbeschränkungen während der Covid-19-Pandemie zwischen 2020 und 2022 haben auch bei Kindern und Jugendlichen zu einer Reduzierung sozialer Kontakte und zu einem Gefühl der Einsamkeit geführt [20]. Diese Erfahrungen verdeutlichen, wie wichtig es ist, dass Kinder früh lernen, soziale Kontakte aufzubauen. Während sich früher Eltern mit kleinen Kindern in der Nachbarschaft und auf Spielplätzen getroffen haben, findet heute der Aufbau sozialer Kontakte vor der Einschulung häufig in Krippen, Kindergärten und Vorschulen statt.

Umfang und Qualität der Kinderbetreuung unterscheiden sich international erheblich. Bei Kindern unter 3 Jahren sind das Angebot und die Nutzung der Kinderbetreuung in nordeuropäischen Ländern wie Dänemark und Schweden mit großem Abstand an der Spitze, gefolgt von Frankreich, Belgien und Luxemburg. Osteuropäische Länder wie Polen und die Tschechische Republik befinden sich am unteren Ende der Länderskala, während Deutschland und Österreich mittlere Plätze einnehmen [21]. Das gut ausgebaute System der Kinderbetreuung erklärt die in Nordeuropa seit Jahrzehnten bestehende hohe Erwerbstätigkeit von Frauen. Beides, die Integration von Frauen in den Arbeitsmarkt und frühe Sozialisationsprozesse in Kinderbetreuungseinrichtungen mit qualifizierten Fachkräften, kann sich positiv auf Einsamkeitserfahrungen auswirken.

### Schulen.

Im Schulalter erfolgt bei Kindern und Jugendlichen der Aufbau von Peer-Groups. Sie bilden Freundeskreise und können die Erfahrung machen, aus Gruppen ausgeschlossen zu werden. Ein sozialer Ausschluss im Jugendalter kann dazu führen, dass Betroffene auch später Schwierigkeiten haben, soziale Beziehungen aufzubauen [22]. Entsprechend wichtig ist es, dass Kinder und Jugendliche in einem sozialen Kontext aufwachsen, der sie stärkt und vor sozialen Ausschlussprozessen schützt. Ein solcher Kontext kann eine Ganztagsschule mit Aktivitäten im Anschluss an den Schulunterricht sein, die durch Pädagog*innen angeleitet werden, die von den Schüler*innen als unterstützend wahrgenommen werden.

Ganztagsschulen gibt es fast durchgehend in Finnland, Großbritannien, Kanada, Neuseeland, Schweden, Südkorea und in den USA [23]. In Finnland bietet die Ganztagsschule einen sozialen Kontext mit einer hohen pädagogischen Qualität und vielen Lehrkräften und weiteren Pädagog*innen je Schüler*in. Eine hohe Qualität der Betreuung kann dazu beitragen, die Entwicklung von Einsamkeit frühzeitig zu erkennen und präventiv einzugreifen. Eine solche Unterstützung kann in Schulen durch Pädagog*innen oder Schulpsycholog*innen erfolgen, wie am Beispiel von Dänemark gezeigt wurde [24]. Während in Deutschland auf eine Schulpsycholog*in fast 10.000 Schüler*innen kommen, sind es in Dänemark, Estland und der Schweiz weniger als 1000 Schüler*innen [25]. An diesem Beispiel ist das unterschiedliche Potenzial zu erkennen, mit geschulten Kräften Kinder und Jugendliche über Einsamkeit und Schutzmaßnahmen zu informieren und sie in Schulen mit protektiven Maßnahmen zu stärken. Als wichtig für die Reduzierung von Einsamkeit in der Schule haben sich eine soziale Unterstützung durch Mitschüler*innen und Lehrer*innen sowie eine als positiv wahrgenommene Lernumgebung erwiesen [26].

### Vereine.

Eine weitere wichtige Institution für soziale Interaktionen sind Vereine. Der Leitgedanke von Vereinen ist es, Menschen für gemeinsame Aktivitäten zusammenzubringen. Unterschiedliche Altersgruppen tragen zum Vereinsleben bei und Mitglieder lernen früh, sich zu beteiligen. Sie erfahren, dass sie oder ihre Kinder durch ehrenamtliche Mitglieder unterstützt werden. Dadurch steigt die Bereitschaft, selbst Aufgaben im Verein zu übernehmen und sich ehrenamtlich zu engagieren. Durch die praktizierte Solidarität wird gelernt, für andere Menschen einzustehen, Teil einer Gruppe zu werden und auf andere Menschen zuzugehen. Menschen, die in jungen Jahren im Verein aktiv waren, suchen auch im höheren Alter den Kontakt zu Menschen im Verein. Dadurch tragen Vereine in allen Altersgruppen zur Reduzierung von Einsamkeit und zu einer Resilienz gegenüber Einsamkeit und den damit verbundenen Folgen bei [27].

Erneut befinden sich die nordeuropäischen Länder an der Spitze. Etwa ein Viertel der Bevölkerung über 14 Jahren ist in Schweden regelmäßig im Sportverein ehrenamtlich aktiv. Es folgen die Schweiz, Dänemark und die Niederlande. Mit Anteilen von 10 % ehrenamtlichen Engagements in Sportvereinen bilden Deutschland und Großbritannien das Mittelfeld. Ost- und südeuropäische Länder wie Ungarn, Spanien, Italien und Polen weisen vergleichsweise niedrige Werte auf [28].

### Gesundheitsversorgung.

Gesundheitsprobleme und Einsamkeit sind eng miteinander verbunden. Ähnlich wie bei Programmen der gesundheitlichen Vorsorge und Prävention kann ein Schutz vor Einsamkeit dazu beitragen, die Gesundheit zu stärken. Für viele Menschen im höheren Alter zählt das Gesundheitspersonal zu den wichtigsten Vertrauenspersonen [14] und die Schwelle, sie anzusprechen, ist durch die Patientenrolle geringer als bei anderen potenziellen Hilfen.

Eine niederländische Studie zeigt, dass Hausärzte*innen die wichtige Bedeutung des Gefühls von Einsamkeit ihrer Patient*innen anerkennen, jedoch Schwierigkeiten haben, zu den entsprechenden Situationen einen ärztlichen Rat zu geben [29]. Für die Erfassung von gesundheitlichen Problemen im Zusammenhang mit Einsamkeit ist es wichtig, dass sich Ärzt*innen und weiteres Gesundheitspersonal Zeit bei der Behandlung nehmen, und dies entspricht auch dem Wunsch der Patient*innen [30]. Während die durchschnittliche Dauer der Kontakte zwischen Hausärzt*in und Patient*in in Belgien und der Schweiz etwa 15 min beträgt, sind es in Großbritannien und den Niederlanden 10 min und in Deutschland und Spanien 7–8 min [31]. Courtin und Knapp [29] sehen es außerdem als wichtig an, das Gesundheitspersonal besser zur Problematik der Einsamkeit und deren krankheitserzeugender Wirkung zu schulen. In einer kanadischen Studie wird betont, dass vor allem Krankenpfleger*innen die Problematik der Einsamkeit berücksichtigen und hierfür ausgebildet werden sollten [29].

In Großbritannien machen viele Hausärzt*innen die Erfahrung, dass täglich Patient*innen aufgrund von Einsamkeit in die Praxis kommen. Es wurden Sozialverschreibungen eingeführt, mit denen Patient*innen vergünstigt an sozialen und kulturellen Aktivitäten teilnehmen können. Bisher konnte jedoch nicht festgestellt werden, ob dadurch Einsamkeit wirksam reduziert wird [32, 33]. Auch wenn in diesem Bereich weitere Forschung erforderlich ist, zeigen diese Beispiele, wie wichtig es ist, im Gesundheitssystem qualifizierte Ansprechpartner*innen für das Problem der Einsamkeit zu etablieren.

### Pflege.

Einsamkeit kann entstehen, wenn Menschen nach einer Erkrankung oder einem Unfall nicht mehr selbstständig in ihrem früheren Zuhause leben können. Bei einem Umzug in ein Alten- oder Pflegeheim, das weiter von ihrem früheren Wohnumfeld entfernt ist, kann sich die Einsamkeit erheblich erhöhen. Oft hängt es von dem Versorgungsprozess im Gesundheitssystem, vor allem im Krankenhaus, und dem Pflegesystem ab, ob und wie ältere Menschen nach einem Krankenhausaufenthalt zu Hause weiterversorgt werden können [34].

Fast alle Menschen haben den Wunsch, im Alter zu Hause in ihrer gewohnten Umgebung zu leben. Die internationale Bezeichnung lautet „Ageing in Place“ [35]. Inzwischen wird in vielen Ländern mit Anpassungen der Wohnumgebung und der sozialen Sicherungssysteme auf diesen Wunsch reagiert [36]. Menschen, die im Alter zu Hause leben, sind in höherem Maße als in Alten- oder Pflegeheimen auf die Unterstützung durch ihre sozialen Netzwerke (Familie, Freund*innen, Nachbar*innen) angewiesen. Dadurch ergeben sich neue Anforderungen an die Nachbarschaften oder Quartiere, in denen ältere Menschen leben. Sie benötigen eine Infrastruktur mit Einkaufsmöglichkeiten und für Pflege‑, Arzt- und Behördengänge sowie weitere soziale Leistungen im näheren Umfeld [37]. Bei Pflegebedürftigkeit hat die Hauskrankenpflege an Bedeutung gewonnen [34]. Sobald Lücken in der Versorgungskette entstehen, erfolgt dennoch häufig ein Umzug in ein Pflegeheim. Neue Konzepte wie „hospital at home“ [38] können dazu beitragen, dass ältere Menschen auch bei einer vorübergehenden intensiven Pflege in ihrem Zuhause wohnen bleiben.

Bei denjenigen, die trotz Pflegebedürftigkeit in ihrem Wohnumfeld wohnen bleiben, wird Einsamkeit vor allem durch den Ausbau der öffentlichen Infrastruktur und ambulanten Hilfen reduziert [39]. Bei einem Wechsel von der gewohnten Umgebung in eine Alten- und Pflegeeinrichtung besteht die Aufgabe darin, soziale Kontakte aufrechtzuerhalten und neue Möglichkeiten für soziale Aktivitäten zu etablieren, um Einsamkeit zu vermeiden [40].

## Direkte Maßnahmen

In diesem Abschnitt werden Beispiele für direkte Maßnahmen dargestellt, die entwickelt wurden, um Einsamkeit und/oder ihre Folgen zu reduzieren. Die Weltgesundheitsorganisation (WHO) und die Vereinten Nationen (UN) haben in den 2020er-Jahren begonnen, umfassend auf die schwerwiegenden Folgen von Einsamkeit und sozialer Isolation hinzuweisen [41]. Einflussreich sind in diesem Zusammenhang der Policy-Brief „Addressing loneliness and social isolation among older people in Europe“ [13] und das Strategiepapier der WHO „Social isolation and loneliness among older people“ [42]. Darin werden u. a. folgende Maßnahmen empfohlen: Förderung und Training sozialer Kompetenzen und des gegenseitigen Kennenlernens, kognitive Verhaltenstherapien, Verbesserung der Infrastruktur sowie Förderung von altersgerechten Gemeinden und Nachbarschaften [42].

### Maßnahmen zur Verhaltensänderung.

Besonders früh wurden Maßnahmen gegen Einsamkeit eingeführt, die das Ziel haben, individuelle Verhaltensänderungen zu fördern. Dieser Fokus wurde vor allem in der Anfangsphase der Entwicklung von Programmen gegen Einsamkeit durch zahlreiche wissenschaftliche Studien gestützt [17, 43]. Maßnahmen, die in den 1970er- und 1980er-Jahren vor allem in den USA eingeführt wurden, waren ursprünglich zur Reduzierung von Angst und Schüchternheit entwickelt worden. Aus dieser Zeit stammen auch Maßnahmen, in denen eine kognitive Verhaltenstherapie mit einem Training sozialer Kompetenzen verknüpft wurde [43]. Insbesondere die kognitive Verhaltenstherapie hat gemäß einer Studie von Masi et al. [43] in diesem Kontext das Potenzial, Einsamkeit zu verringern. In einer Studie, die 30 Interventionsanalysen von 1979 bis 2002 umfasst, kommen Cattan et al. [2] zu dem Schluss, dass Gruppeninterventionen mit Beteiligungsmöglichkeiten, die mit einem umfassenden Bildungskonzept kombiniert werden, sowie auf spezifische Zielgruppen ausgerichtete Maßnahmen (z. B. Frauen, Verwitwete, Personen mit eingeschränkter psychischer Gesundheit) besonders erfolgreich sind. Weitere Studien zeigen positive Effekte von Programmen zur Förderung der Achtsamkeit und der sozialen Unterstützung [3].

### Maßnahmen für ältere Menschen.

Dadurch, dass sich erste Hinweise eines Anstiegs der Einsamkeit bei älteren Menschen zeigten und die im Alter kleineren sozialen Netzwerke diese Befürchtungen stützten, waren erste Maßnahmen gegen Einsamkeit auf diese Zielgruppe ausgerichtet.

In Großbritannien ist AGE UK die größte karitative Organisation, die das Ziel hat, die Lebenssituation im höheren Alter zu verbessern. In dem Report „Campaign to End Loneliness“ werden insbesondere personenbezogene Maßnahmen für ältere Menschen empfohlen [33]. Ein wichtiges Instrument ist ein schriftlicher Leitfaden, mit dessen Hilfe in einem Gespräch mit älteren Menschen Lösungen für ihre durch Einsamkeit geprägte Situation identifiziert werden sollen. Parallel zu diesen meist telefonischen Gesprächen sollen Sozialarbeiter*innen über persönliche Kontakte Vertrauen aufbauen und soziale Aktivitäten fördern. Als ein Nachteil dieses auf individuelle Bedürfnisse zugeschnittenen Programms werden hohe Kosten und Komplexität hervorgehoben [44]. In Berichten über Erfahrungen mit diesen Maßnahmen wird betont, dass ältere Menschen bei der Entwicklung von Programmen gegen Einsamkeit für eine bessere soziale Integration und ein positives Selbstwertgefühl beteiligt werden sollten [44]. In Kanada werden in dem „Keeping-Connected“-Programm zunächst telefonisch die Risikofaktoren und der Grad der Einsamkeit bei älteren Menschen erhoben. Anschließend werden regelmäßig Telefongespräche geführt (1- bis 3‑mal pro Woche für 20–30 min). Gesprächsthemen sind die Familie, aktuelle politische und gesellschaftliche Themen, Informationen über Krankenhäuser und ambulante Pflegedienste, Anleitungen bei der Nutzung von technischen Geräten oder Kochrezepte.^1^

In Neuseeland und in den USA wurden Programme mit einem Assessment von Einsamkeit im Rahmen der Pflege eingeführt [29]. In Australien wurde zunächst der Bedarf an Gesundheits- und Pflegeleistungen erhoben. Auf dieser Grundlage erhielten Pflegekräfte eine Schulung und eine Liste mit Instruktionen zur Verbesserung der körperlichen Funktionen, damit ältere Menschen am gesellschaftlichen Leben teilnehmen können [29]. In Frankreich wurde mit MONALISA^2^ ein Programm zur gegenseitigen Unterstützung etabliert, das vor allem auf die Reduzierung von Einsamkeit und sozialer Isolation älterer Menschen ausgerichtet ist. Bürger*innen können sich in 850 Teams online registrieren und ehrenamtliche Aufgaben übernehmen. Es geht um den Aufbau gegenseitiger Unterstützungsleistungen auf lokaler Ebene, in die Freiwillige, Familienmitglieder und professionelle Kräfte aus der sozialen Arbeit und der Gesundheitsversorgung und Pflege einbezogen werden [13].

Den Kern des KISS-Programms^3^ in der Schweiz bildet ein Zeitvorsorgemodell [13]. Bei ehrenamtlichem Engagement wird ein KISS-Zeitnachweis erworben, den man nutzen kann, wenn man selbst auf Leistungen angewiesen ist. Es werden Tandems gebildet, die sich gegenseitig unterstützen und für ihre Aufgaben durch Fachkräfte geschult werden. Dadurch soll ermöglicht werden, dass ältere Menschen länger in ihrem Lebensumfeld wohnen und bestehende Kontakte aufrechterhalten können. Die Vermeidung stationärer Pflege wird als Schutz vor Einsamkeit angesehen [13]. Im Unterschied zu anderen Programmen wird im KISS-Programm mit Anreizen bzw. Belohnungen gearbeitet, die über intrinsisch motivierte Solidaritätsbeziehungen hinausgehen. Die Stärkung von Generationsbeziehungen und von zivilgesellschaftlichen Aktivitäten soll zu einer hohen Lebensqualität und einer Reduzierung von Einsamkeit im Alter beitragen.

### Freundschaftsprogramme.

Als besonders erfolgreich haben sich in mehreren Ländern Freundschaftsprogramme erwiesen. Sie sind ebenfalls vor allem auf Ältere ausgerichtet, können jedoch auch andere Altersgruppen einbeziehen. Ein Programm, das regelmäßig evaluiert wird, sind die finnischen „Circle of Friends“ mit mehr als 1200 Gruppen und über 12.000 Teilnehmer*innen. Es werden Treffen für soziale Kontakte etabliert und über Empowerment-Kurse das Selbstbewusstsein der Mitglieder und der soziale Austausch innerhalb der Gruppen gefördert. Wissenschaftliche Evaluationen dieses Programms zeigen, dass dadurch neue Freunde gefunden werden, das individuelle Wohlbefinden steigt und in Pflegeeinrichtungen die empfundene Einsamkeit sinkt [13].

In den Niederlanden werden in den „Buurtcirkel Neighbourhood Circles Netherlands“^4^ Gruppen in der Nachbarschaft durch professionelle Kräfte gecoacht und von Wohlfahrtsorganisationen und den Kommunen unterstützt. Über eine Homepage können Interessierte Kontakt zu den Gruppen aufnehmen. Über diese aus 9 bis 12 Mitgliedern bestehenden Gruppen sollen Menschen zusammengeführt, gegenseitige Hilfen aufgebaut und Einsamkeit reduziert werden [13].

In Irland ermöglicht ALONE^5^ eine Anbindung von Freundschaftsprogrammen an Wohn- und Pflegemöglichkeiten im Alter. Dabei werden Freundschaftsnetzwerke aufgebaut, in denen ältere Menschen durch Besuche und Anrufe unterstützt werden und ein Leben zu Hause ermöglicht werden soll. Die Basis für diese Netzwerke und für eine Einbindung in das lokale Umfeld bilden altersgerechte Wohnungen, die durch ALONE zur Verfügung gestellt und mit weiteren professionellen und ehrenamtlichen Hilfen für einsame und isolierte Menschen kombiniert werden [13].

### Universelle Maßnahmen.

Als universelle Maßnahmen werden hier Programme bezeichnet, die in einer allgemeinen Form und ohne eine spezielle Zielgruppe darauf ausgerichtet sind, Einsamkeit zu reduzieren. Es geht nicht darum, einsame Menschen zu identifizieren und ihnen Hilfen anzubieten. Bei universellen Maßnahmen handelt es sich häufig um Programme, mit denen die Bedingungen in der Nachbarschaft verbessert werden. Ein wichtiges Element von Konzepten auf Gemeinde- oder Nachbarschaftsebene sind Veränderungen der Infrastruktur, einschließlich öffentlicher Plätze, und von Gebäuden. Bei der Entwicklung dieser Maßnahmen werden Anwohner*innen häufig aktiv einbezogen, um das soziale Engagement, den Zusammenhalt und eine positive Haltung gegenüber den Veränderungen zu fördern. Diese als Neighbouring (Nachbarschaftsstärkung) bezeichneten Maßnahmen werden sehr positiv evaluiert. Sie sollen dazu beitragen, die Identifizierung mit der Gemeinde, dem Stadtteil bzw. der Nachbarschaft zu stärken und das Risiko der Einsamkeit zu reduzieren [45]. Die Identifikation hängt in hohem Maße von dem Gefühl der Sicherheit und von einer positiven Bewertung der Nachbarschaft ab. Das Gefühl der Sicherheit sinkt, wenn soziale Probleme, Müll und Vandalismus wahrgenommen werden. Dann steigt das Misstrauen gegenüber anderen Menschen und die Bereitschaft für soziales Engagement sinkt. Als Folge steigt die empfundene Einsamkeit [45].

In Großbritannien haben Nachbarschaftsnetzwerke den Sinn für Gemeinschaft gestärkt. Menschen, die in öffentlichen Wohnungen leben, bauen bei einer stärkeren Identifizierung mit ihrer Nachbarschaft häufiger Kontakte zu ihren Nachbar*innen auf, um gemeinsam Probleme in der Nachbarschaft zu lösen [45]. Ein Projekt in den Niederlanden zeigt ebenfalls, dass Menschen, die sich mit ihrem Stadtteil identifizieren und sich zugehörig fühlen, die sozialen Netzwerke in der Nachbarschaft stärken und seltener einsam sind [46].

Viele dieser Konzepte bleiben auf der lokalen Ebene verankert. Demgegenüber hat Australien mit dem „Neighbour Day“ (Nachbarschaftstag) eine nationale Strategie gegen Einsamkeit etabliert. Es handelt sich um ein Programm, das auf lokaler Ebene durchgeführt wird, um die soziale Identifikation der Menschen mit ihrer Nachbarschaft zu stärken [45]. Dadurch, dass dieser Nachbarschaftstag landesweit immer am letzten Sonntag im März stattfindet, ist dieses Programm gegen Einsamkeit in den Medien sehr präsent und das Ziel der Stärkung des sozialen Zusammenhalts und des Wohlbefindens zur Vorbeugung vor Einsamkeit erhält immer mehr Unterstützung in der Bevölkerung und durch politische und gesellschaftliche Akteure [45].

### Digitale Maßnahmen.

Studien zum Einfluss von digitalen Maßnahmen zeigen, dass sie vor allem dann eine positive Wirkung haben und Einsamkeit reduzieren, wenn sie Programme zur Förderung eines direkten persönlichen Austauschs ergänzen [16, 47]. Mehrere Konzepte sind darauf ausgerichtet, die Mitwirkung an Aktivitäten in der Nachbarschaft über IT und soziale Medien zu stärken. Auch der australische Nachbarschaftstag wird mithilfe sozialer Medien im Bewusstsein der Menschen verankert [45]. Erfolgreiche Beispiele sind Internetplattformen, die Menschen auf lokaler Ebene verbinden, wie in den USA das „Social Isolation and Loneliness Outreach Toolkit“ oder die „Connect 2Tools to Overcome Social Isolation“ [45].

Bei E‑Interventionen gegen Einsamkeit wird z. T. davon ausgegangen, dass Ältere nicht über das Know-how verfügen, um Online-Plattformen, Informationen und Kontaktmöglichkeiten über das Internet bzw. soziale Medien nutzen zu können. Entsprechend wurde z. B. in dem niederländischen Esc@pe-Projekt älteren Menschen ein Zugang zu Computer und Internet in Verbindung mit Schulungen ermöglicht, um die Kommunikationsmöglichkeiten des Internets nutzen zu können. Bei einer Evaluation dieses Programms konnte eine signifikante Reduzierung der Einsamkeit bei höheren Bildungsgruppen festgestellt werden [48].

## Ausblick: Maßnahmen ausbauen und verbessern

Interventionen, die das Wissen über Einsamkeit und die Kompetenzen im Umgang mit Einsamkeit verbessern, zeigen i. d. R. positive Effekte [2]. Untersuchungen des Nachbarschaftstags in Australien haben ergeben, dass dadurch die Identifikation mit der Nachbarschaft und der soziale Zusammenhalt gestärkt werden [45]. Außer diesen wichtigen direkten Maßnahmen zur Reduzierung von Einsamkeit ist zu berücksichtigen, dass der soziale Kontext und bestehende gesellschaftliche Institutionen einen bedeutenden Schutz vor Einsamkeit bieten. Die Studie von Nyqvist et al. [49] zeigt, dass Wohlfahrtsstaaten, die einen Schutz vor Armut und weiteren sozialen Krisensituationen bieten, besonders gut vor Einsamkeit schützen. Die steigende Einsamkeit bei jüngeren Altersgruppen [50] ist allerdings ein Hinweis, dass zusätzliche Programme gegen Einsamkeit erforderlich sind.

Um Kinder und Jugendliche vor Einsamkeit zu schützen, sind die Voraussetzungen in Kinderbetreuungseinrichtungen, Schulen und Vereinen zu verbessern. Länder, die über eine hohe Zahl an Pädagog*innen an Schulen verfügen, weisen häufig geringere Einsamkeitswerte bei Kindern und Jugendlichen auf. In Sportvereinen ist eine hohe Qualifikation der Trainer*innen erforderlich, um Kinder und Jugendliche in soziale Gruppen zu integrieren und so zu stärken, dass sie sich bei sozialen Ausschlusserfahrungen nicht zurückziehen. Hierfür sind neben den vielen Ehrenamtlichen auch professionelle Trainer*innen erforderlich, um neue Aufgaben wie einen Schutz vor Einsamkeit übernehmen zu können. Es ist wichtig, Angebote für ältere Menschen in Vereinen anzubieten und ihnen dadurch regelmäßige soziale Kontakte zu ermöglichen.

Ältere Menschen haben den Wunsch, so lange wie möglich in ihrem Zuhause und dem gewohnten sozialen Umfeld zu leben. Wenn dies gelingt und die Bedingungen für soziale Kontakte verbessert werden, wird ein Schutz vor Einsamkeit geschaffen, der den Wünschen älterer Menschen entspricht. Das Ziel, im Alter zu Hause zu leben, kann häufig nicht umgesetzt werden, wenn nach einem Krankenhausaufenthalt eine Anschlussversorgung zu Hause nicht sichergestellt werden kann. Eine auf die Bedürfnisse und Wünsche älterer Menschen zugeschnittene Hauskrankenpflege und eine enge Abstimmung zwischen Krankenhausversorgung und Hauskrankenpflege tragen dazu bei, dass ältere Menschen in ihrem gewohnten sozialen Umfeld leben bleiben können. Hierfür ist eine Koordination von Gesundheits- und Pflegeleistungen erforderlich, die in den Niederlanden und in Skandinavien häufig durch die Kommune übernommen wird [34].

Neu in den Fokus geraten ist die hohe Bedeutung der Nachbarschaft. Angebote für soziale Kontakte sind dort wichtig, wo die Menschen leben. Es geht darum, das Gefühl der Sicherheit in dem jeweiligen Stadtteil zu erhöhen. Öffentliche Plätze, Parks und Begegnungsstätten sind für alle Altersgruppen für soziale Kontakte wichtig. Bei Älteren ist zu gewährleisten, dass diese Orte erreichbar sind [46]. Dadurch können das Zugehörigkeitsgefühl und die sozialen Netzwerke in der Nachbarschaft verbessert und Einsamkeit wirksam bekämpft werden. Die Etablierung lokaler Freundeskreise hat sich ebenfalls als erfolgreiche Strategie gegen Einsamkeit erwiesen. Ähnlich wie bei Nachbarschaftsprogrammen haben Freundeskreise den Vorteil, dass einsame Menschen sich nicht als passive Leistungsempfänger wahrnehmen, sondern den Schutz vor Einsamkeit aktiv mitgestalten.

## Footnotes

1 https://www.closingthegap.ca/.

2 https://www.monalisa-asso.fr/.

3 https://fondation-kiss.ch/.

4 https://buurtcirkel.nl/.

5 https://alone.ie/.
